# Computational Nanotoxicology Models for Environmental Risk Assessment of Engineered Nanomaterials

**DOI:** 10.3390/nano14020155

**Published:** 2024-01-10

**Authors:** Weihao Tang, Xuejiao Zhang, Huixiao Hong, Jingwen Chen, Qing Zhao, Fengchang Wu

**Affiliations:** 1 National-Regional Joint Engineering Research Center for Soil Pollution Control and Remediation in South China, Guangdong Key Laboratory of Integrated Agro-Environmental Pollution Control and Management, Institute of Eco-Environmental and Soil Sciences, Guangdong Academy of Sciences, Guangzhou 510650, China; 2 Key Laboratory of Pollution Ecology and Environmental Engineering, Institute of Applied Ecology, Chinese Academy of Sciences, Shenyang 110016, China; 3 National Center for Toxicological Research, U.S. Food and Drug Administration, 3900 NCTR Rd., Jefferson, AR 72079, USA; 4 Key Laboratory of Industrial Ecology and Environmental Engineering (Ministry of Education), Dalian Key Laboratory on Chemicals Risk Control and Pollution Prevention Technology, School of Environmental Science and Technology, Dalian University of Technology, Dalian 116024, China; 5 State Key Laboratory of Environmental Criteria and Risk Assessment, Chinese Research Academy of Environmental Sciences, Beijing 100012, China

**Keywords:** engineered nanomaterials, computational nanotoxicology, exposure models, hazard models

## Abstract

Although engineered nanomaterials (ENMs) have tremendous potential to generate technological benefits in numerous sectors, uncertainty on the risks of ENMs for human health and the environment may impede the advancement of novel materials. Traditionally, the risks of ENMs can be evaluated by experimental methods such as environmental field monitoring and animal-based toxicity testing. However, it is time-consuming, expensive, and impractical to evaluate the risk of the increasingly large number of ENMs with the experimental methods. On the contrary, with the advancement of artificial intelligence and machine learning, in silico methods have recently received more attention in the risk assessment of ENMs. This review discusses the key progress of computational nanotoxicology models for assessing the risks of ENMs, including material flow analysis models, multimedia environmental models, physiologically based toxicokinetics models, quantitative nanostructure–activity relationships, and meta-analysis. Several challenges are identified and a perspective is provided regarding how the challenges can be addressed.

## 1. Introduction

Nanotechnology is regarded as a key enabling technology owing to its potential to contribute to societal well-being and economic growth across industrial sectors [1]. The global nanotechnology market already reached $39.2 billion in 2016 and is expected to reach $125 billion by 2024 [2,3]. In addition, billions of dollars were invested from global public funding in research and development of nanotechnology during the last decade [4]. Nanotechnology already has been applied in many different sectors such as agriculture, cosmetics, electronics, environment, food, medicine, printing, renewable energies, textile, and others [5]. The nanotechnology industry currently consists of 10,150 marketable nano-enabled products produced by 3246 companies from 64 countries (https://product.statnano.com, accessed on 1 November 2023). In 2010, the estimated global production of engineered nanomaterials (ENMs) varied from 268 to 318 thousand metric tons, increasing at a rate of approximately 25% per year [6]. The term “ENMs” used in the review includes engineered nanoparticles, nanofibers, nanoplates, quantum dots, and other nanostructured surfaces [7].

ENMs can be released into the environment during their whole life cycle such as production, manufacture, use, and disposition. They are defined as novel entities included in the planetary boundaries, which aim to identify a “safe operating space” to develop and thrive for human societies [8,9,10]. In order to identify a “safe operating space” for ENMs, the environmental and health risks of ENMs should be taken into account, including assessing the exposure and hazards of ENMs [11,12,13]. The exposure and hazard level can be obtained by field monitoring or in vitro and vivo assays [7,14,15,16]. However, these methods are expensive and time consuming, making them impractical to assess the exposure and hazards of the increasing number of ENMs [17]. For example, more than 500 animals and $4 million are required to test a single chemical with the 2-year rat carcinogenicity assay [18,19]. Additional animals and money are needed for the follow-up experiments to elucidate toxicokinetics and mechanisms of toxicity [20]. Thus, rapid and efficient methods are needed to assess the risk of ENMs.

Computational toxicology is an alternative approach to experimental methods and has been widely used in risk assessment research [21,22,23]. Computational toxicology is an interdisciplinary combination of environmental chemistry, physical chemistry, biochemistry, toxicology, bioinformatics, chemoinformatics, statistics, computer science (e.g., artificial intelligence, machine learning), and many other relevant subjects [24]. In computational toxicology, external exposure concentrations (also known as environmental concentrations) of chemical substances can be estimated with environmental fate and transport models such as material flow analysis models and multimedia environmental models [25]. Although external exposure concentrations are traditionally used to quantify the toxicity effects of chemicals in risk assessment, internal exposure concentrations are more suitable for understanding the toxicity effects of chemicals. In order to predict internal exposure concentrations, physiologically based toxicokinetics (PBTK) models are particularly used to quantitatively predict the absorption, distribution, metabolism, and excretion (ADME) processes of chemicals in organisms which in turn correlate the external exposure concentrations with the internal exposure concentrations (target concentrations) [26,27]. Quantitative structure–activity relationship (QSAR) models have been widely used for predicting the hazards of chemicals [28]. With the advancement of artificial intelligence, improvement in computing hardware, and increasing data volumes, various machine learning and deep learning technologies have been adopted in QSAR modeling, which makes QSAR a core method in computational toxicology methodologies [29,30,31].

Computational toxicology methods can also be employed in evaluating the exposure and hazards of ENMs, which is known as computational nanotoxicology [32]. Assessing the exposure and hazards of ENMs is more challenging than assessing the exposure and hazards of chemicals. The size of ENMs is in the nanoscale with at least one dimension < 100 nanometers. With the small size, lots of properties such as particle density and surface area are different from their bulk form [33]. These unique properties of ENMs play important roles in the transport, fate, and reactivity of ENMs in the environment and are the critical attributes associated with nanotoxicity [34,35]. In addition, the physicochemical properties of ENMs such as surface coatings, solubility, and surface charge can affect their behavior in biological systems, leading to altered exposure, toxicokinetics, and toxicity [36].

**Figure 1:**
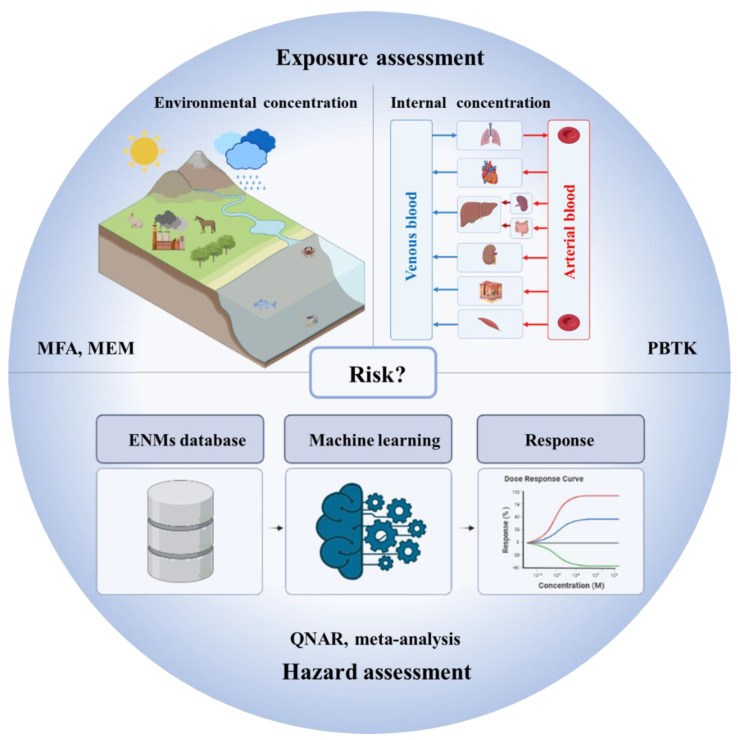
Environmental risk assessment framework for ENMs with computational nanotoxicology models (ENMs: engineered nanomaterials, MFA: material flow analysis, MEM: multimedia environmental models, PBTK: physiologically based toxicokinetics models, QNAR: quantitative nanostructure–activity relationships). The picture was created with BioRender.com.

The complex properties of ENMs make it a big challenge to develop computational nanotoxicology models for assessing the exposure and hazards of ENMs. To promote computational nanotoxicology, this review summarizes some recent key advances in computational nanotoxicology, including material flow analysis models, multimedia environmental models, physiologically based toxicokinetics models, quantitative nanostructure–activity relationships, and meta-analysis. As some reviews have elaborated on the methodologies of computational nanotoxicology models [37,38], this review focuses on their applications and challenges in environmental risk assessment. Figure 1 depicts the environmental risk assessment framework for ENMs with computational nanotoxicology models.

## 2. Literature Survey

A literature review was conducted with the Web of Science databases. For material flow analysis and multimedia environmental models, the topic terms “nanomaterial fate model” and “nanomaterial material-flow model” were employed for selecting research from 2016 to 2023, as several vital reviews were published up to 2016 [39,40]. For physiologically based toxicokinetics models, the topic terms “(PBPK or PBTK) and nano*” were employed for selecting research from 2018 to 2023 as several vital reviews were published by 2018 [41,42]. For quantitative nanostructure–activity relationships, the topic terms of “Nano-QSAR” were employed for selecting research from 2017 to 2023 as several vital reviews were published by 2017 [43,44]. For the meta-analysis, the topic terms “(nanoparticle or nanomaterial) and toxic* and meta-analysis” were employed for selecting research from 2001 to 2023. In total, 546 articles were retrieved and we further read these articles to filter articles that are not relevant to the review topic.

## 3. Material Flow Analysis and Multimedia Environmental Models

Environmental exposure models are indispensable tools for assessing the exposure of chemicals in the environment. Exposure models are particularly useful for assessing the exposure of ENMs because the detection of ENMs in environmental media is challenging and suitable analytical technology remains under development [45]. Material flow analysis (MFA) and multimedia environmental model (MEM) are two types of exposure models that can model the fate of ENMs and predict their concentrations in the environment.

MFA tracks stock and flows of materials into and between environmental compartments (e.g., sediment, water, soil, air) and technological compartments (e.g., landfills, incinerators) during stages of production, manufacturing, use, and final disposal. It is the earliest method to model the fate of ENMs. To develop MFA models, one should first determine model structures and mathematical modeling approaches (e.g., probabilistic or deterministic). The model structure can be applied in a specific case by determining the system boundary. The necessary input parameters in an MFA model include production volume within the system boundary, distribution of mass to product categories, release from products, transfer factors for technical compartments, transfer factors during recycling, and transfer factors for environmental compartments [46].

Mueller and Nowack [47] developed the classical MFA model to quantify releases of nano Ag, nano TiO_2_, and carbon nanotubes (CNT) from products in Switzerland. The technological compartments in this model contain waste incineration plants, landfills, and sewage treatment plants, while the environmental compartments include air, water, and soil. The predicted environmental concentrations (PEC) of the three nanoparticles were calculated based on single-value input parameters. The results revealed that the PEC values of nano-TiO_2_ in water (0.7–16 µg/L) are close to or higher than the predicted no-effect concentrations of nano-TiO_2_ (<1 µg/L), indicating nano TiO_2_ can pose risks to aquatic organisms.

The classical MFA models only employed singe-value parameters as model inputs and cannot account for the inconsistency and variability of the constructed models. To address this issue, probabilistic material flow analysis (PMFA) models were developed by considering all possible model inputs [48]. In a PMFA model, model input parameters were represented by probability distributions derived from empirical data or expert judgment [49]. PMFA models were employed to predict country-specific emissions of nano-Ag, nano-TiO_2_, and nano-ZnO throughout the life cycle within Europe [50]. Monte Carlo and Markov Chain Monte Carlo simulations were employed to generate the model input and output distribution. The results of the PMFA models demonstrated that nano Ag, nano TiO_2_, and nano ZnO may pose risks to aquatic organisms in sewage treatment effluents in Switzerland, Europe, and the U.S. and that nano-Ag can also produce risks to surface waters. ENMs released into the environment can undergo diverse physical and chemical transformations that may change their forms. Adam et al. [51] developed a PMFA model to quantify the proportions of ENMs (i.e., nano-Ag and nano-TiO_2_) in different forms including pristine, dissolved, transformed, product embedded, and matrix embedded. The recently developed PMFA models have taken into account particle size distributions to determine the amount of nanoscale TiO_2_ pigments released into the environment [52].

Dynamic material flow analysis (DMFA) models can consider time-dependent processes on the use and release of ENMs [53]. DMFA models track flows across a period of time instead of one year and no longer require the assumption that all produced ENMs are immediately released and subsequently distributed to the environmental compartments in one year. Recently, a dynamic probabilistic material flow analysis (DPMFA) method was proposed by combining the advantages of DMFA and PMFA [54]. DPMFA models were developed for predicting the flows of four ENMs (nano-TiO_2_, nano-ZnO, nano-Ag, and CNT) to the environment and quantifying their amounts in the in-use stock and final environmental sinks using two diverse assumptions on shares of ENMs in different products [55,56]. Rajkovic et al. [57] revised the DPMFA model by adding temporal variations of all flows to estimate the fate of four ENMs (CNTs, nano-Ag, nano-TiO_2_, and nano-ZnO) in European waste treatment systems. Another study predicted flows and release concentrations of nano-CeO_2_, nano-Al_2_O_3_, nano-SiO_2_, nano-iron oxides, and quantum dots in European regions with DPMFA models [58].

MFA models were often employed to quantify the release of substances to the environment but were seldom used to model the fate of substances in the environment. Although a few MFA models described the amounts of material flows from one environmental compartment into another based on transfer factors, the transfer factors were derived from observations rather than mechanistic processes [46]. In contrast, MEM models can be used to model behaviors and environmental concentrations of chemical substances in environmental compartments based on mechanistic descriptions of fate processes. In MEM models, interphase mass transport rates of chemicals in gaseous and dissolved phases are governed by fugacity (chemical potential) driving forces that are constrained by thermodynamic equilibriums. This does not agree with the transport behavior of ENMs in the environment which is governed by physical transport mechanisms of particulate matter. Thus, the rates of intermedia transport processes for ENMs (e.g., dry/wet deposition) are governed by particle size distributions.

Recently, some novel MEM models were developed for predicting the environmental concentrations of ENMs. nanoFate [59] is a dynamic MEM model and predicts the time-dependent accumulations of ENMs (such as nano CuO, TiO_2_, ZnO, and CeO_2_) across environmental media. This model included a larger range of ENM processes than some classical MEM models of ENMs such as MendNano [60] and SimpleBox4Nano [61] and considered the long-term accumulation of dissolved metal ions. Based on the nanoFate model, Parker et al. [62] investigated the variation in regional risk of nano-TiO_2_ in six watersheds (New York, Los Angeles, Des Moines, London, Rome, and Zurich). As there are uncertainties in emission estimations and physicochemical properties of ENMs, Meesters et al. [63] evaluated the uncertainty in predicting environmental concentrations of nano-CeO_2_, nano-TiO_2_, and nano-ZnO with the SimpleBox4Nano model to validate the robustness of novel MEM models of ENMs. The result showed that the major source of uncertainty in predicting the environmental concentration of ENMs was associated with production. Table 1 summarized the recent MFA and MEM models for ENMs.

## 4. Physiologically Based Toxicokinetics Models

Once a chemical enters into an organism, the chemical may undergo ADME processes in the organism. PBTK models are useful tools to model the processes of chemicals in organisms. PBTK models are constructed based on the anatomy and physiological structures of living systems, with organs or tissue interconnected via the blood circulation system. In a simulated living system, mass transport processes such as distribution between blood and tissue, cellular uptake, and clearance can be described with mathematical equations based on chemical- and species-specific parameters. The process to develop PBTK models includes (1) specifying general model structures; (2) defining model equations based on an understanding of ADME characteristics; (3) parameterizing models; (4) simulations and/or parameter estimation; and (5) model validation and optimization [41,64]. Detailed introductions on developing PBTK models can be found in other reviews [37,42,65].

Initially, PBTK models were developed for predicting the pharmacokinetics (toxicokinetics) of small molecules such as pharmaceuticals. Simulating the toxicokinetics of ENMs is more challenging than small molecules due to the complex transport mechanisms of ENMs in the living system such as opsonization, uptake of phagocyte system, enzymatic degradation, and changed physicochemical properties or forms. Although some PBTK models were developed for ENMs such as quantum dots [66], nano silver [67], nano gold [68], nano TiO_2_ [69], nano cerium dioxide [70], and nano zinc oxide [71], most of these models were built for intravenously administered ENMs, an exposure route that is unlikely to occur in the environment. Thus, these models are not applicable to the environmental risk assessment of ENMs. However, some of these models still can provide a reference to develop PBTK models of ENMs for environmental risk assessment.

Kumar et al. [72] suggested a PBTK model framework that incorporates major biological processes involved in the biodistribution of nanoparticles. In addition to the liver, spleen, heart, lungs, kidney, and gut compartments, the framework contains a lymph compartment, which plays a critical role in nanoparticle circulation. There are two mechanisms by which ENMs can transport into each compartment, i.e., permeability limited and perfusion limited [73]. The permeability-limited model assumed that there could be a membrane at the capillary or cellular membrane, or both, while the perfusion-limited model assumed that ENMs transportation into tissues is fast, and equilibrium between tissue and blood can be reached instantly.

A common practice for PBTK modeling is to extrapolate existing PBTK models (e.g., rats) for other species (e.g., humans). Through extrapolating the parameter values of validated models, the model can describe the physiology and translocation processes in the extrapolated species. For example, Gakis et al. [74] conducted an interspecies extrapolation from rats/mice to humans with PBTK models to predict the biodistribution of inhaled gold nanoparticles.

As in vivo data for developing PBTK models of ENMs were scarce, Dubaj et al. [75] intended to employ in vitro data to predict the in vivo distribution of polyethylene glycol-coated gold nanoparticles. Human cell lines from different tissues such as TH1, A549, Hep G2, and 16HBE were selected to derive pharmacokinetic parameters of relevant tissues, and the parameters were subsequently employed for PBTK simulation in vivo. The results showed that there were notable differences in the internalized amount of gold nanoparticles between cell lines and the corresponding tissues in vivo, which may be ascribed to the structure difference between tissues and cell lines that lack the natural barriers provided by capillary walls.

The in vivo dose–response relationships can also be extrapolated by in vitro relationships with the PBTK model. Cheng et al. [76] performed an integrated and probabilistic risk assessment of gold nanoparticles by using a human PBTK model to quantify internal concentrations in skin, kidney, liver, and venous plasma. Based on the PBTK model, in vivo dose–response relationships can be predicted with in vitro data. The result showed that the estimated human equivalent doses of gold nanoparticles coated with branched polyethylenimine associated with 5% cell death in the liver and kidney were around 1 and 3 mg/kg, respectively.

Cellular endocytosis is an important process to influences the biodistribution of ENMs. Several studies tried to simulate the process of ENMs in PBTK models. For example, Liang et al. [77] developed a PBTK model for predicting the in vivo biodistribution of quantum dots which are long-circulating ENMs. The structure of this PBTK model included five organ compartments, i.e., lung, spleen, kidney, liver, and the rest of the body. Each compartment was divided into three subcompartments, i.e., tissue, vascular space, and phagocytic cells. The Hill function was used to calculate the uptake rate constant of ENMs by phagocytic cells. However, the studies only considered the endocytosis mechanism in tissues, which ignored the effect of phagocytic cells in blood. Deng et al. [78] tried to investigate the role of the endocytosis mechanism both in the tissue and blood, yet the result revealed that few gold nanoparticles were captured by the phagocytic cells in the blood.

For small molecules, multi-route PBTK models are constructed with a traditional route-to-route extrapolation approach. The extrapolation uses the parameters obtained from one administration route (such as the intravenous route) as the parameters for building PBTK models of other routes (e.g., intratracheal instillation). As ENMs can be covered by different biomolecules (such as proteins) from different administration routes, forming different biomolecular coronas that make the physicochemical properties of ENMs different, the method used for small molecules may not be appropriate for ENMs. Chou et al. [79] built a multi-route PBTK model for gold nanoparticles of different sizes (1.4–200 nm) in adult rats with a method based on route-specific data. In their method, QSAR models were employed for predicting route-specific biodistribution parameters based on the physicochemical properties of gold nanoparticles (e.g., size, surface area, and Zeta potential). Their study demonstrated that the route-specific data were more suitable for developing multi-route PBTK models of ENMs than the traditional route-to-route extrapolation approaches. In addition to exposure route, dose and particle size can also affect the biokinetics of ENMs [70]. Rosário et al. [80] investigated the effects of particle size on the tissue distribution of silver nanoparticles in mice with PBTK models. The results found that smaller Ag nanoparticles (5 nm) are more likely to accumulate in tissues than bigger Ag nanoparticles (50 nm). Table 2 summarized the recent PBTK models for ENMs.

## 5. Quantitative Nanostructure–Activity Relationships

With the advancement of artificial intelligence, quantitative nanostructure–activity relationships (QNAR) have received interest worldwide [81]. As most artificial intelligence methods such as machine learning are data-driven methods, high-quality data are a prerequisite to building reliable QNAR models. Data collection is a laborious process for ENMs as most nanotoxicity data are stored as text documents such as scientific publications, patents, and conference reports, from which researchers manually extract the data or information. The extracted data with proper curation can be assimilated into existing databases, which can promote the model development of ENMs. Some publicly available nanotoxicity databases can be found in other reviews [82].

In QNAR models, nanostructures are mainly described by molecular descriptors, which are calculated from nanostructures with computational software. Traditional QSAR models for chemicals are developed with molecular descriptors calculated from SMILES (simplified molecular input-line entry systems) codes [83]. In the case of ENMs, the molecular descriptors are not suitable for QNAR modeling as different ENMs (e.g., different sizes, coating) are represented by the same SMILES code. The Quasi-SMILES is an expansion of the SMILES code, which can reflect different circumstances and conditions [84]. The Quasi-SMILES descriptors have been employed for predicting the solubility of fullerenes C60 and C70 [85], cytotoxicity of multiwalled carbon nanotubes [86], cell viability of metal oxide nanomaterials [87], and immobilization response of daphnia magna exposed to metal-based ENMs [88].

Simple periodic table-based descriptors are alternatives to quantum-chemical descriptors for QNAR models of ENMs [89]. According to the descriptor calculation method, the periodic table-based descriptors can be divided into two generations. Roy et al. [90] employed the first generation of periodic table-based descriptors to develop QNAR models for predicting the cytotoxicity of heterogeneous TiO_2_-based ENMs. De et al. [91] developed single-species QNAR and interspecies QNAR based on the second generation of periodic table-based descriptors in order to understand the relationship between toxicities against different species and metal oxide nanoparticles along with identifying the major toxicity mechanisms.

Wang et al. [92] built a virtual gold nanoparticle library based on experimental characterization results with an in-house program coded in Python 3.5. A large set of nanodescriptors was calculated by precise surface chemistry simulations of virtual gold nanoparticles. QNAR models for predicting cellular uptake were constructed using 29 selected nanodescriptors and an algorithm of *k* nearest neighbors. The correlation between model predictions and experimental results achieved *R*^2^ values of 0.918 and 0.919 for A549 and HEK293 cells, respectively.

Molecular dynamics (MD) simulations are another method to derive computational descriptors of nanoparticles. Chew et al. [93] modeled 154 self-assembled monolayer-protected gold nanoparticles in aqueous solution with atomistic MD simulations and derived 15 uncorrelated descriptors. The derived descriptors were employed to train QNAR models with LASSO (least absolute shrinkage and selection operator) and random forest regression algorithms for predicting cellular uptake, zeta potentials, and octanol–water partition coefficients of gold nanoparticles. Of the 154 gold nanoparticles, 20% were held for testing models, while the remaining 80% were used for 5-fold cross-validation. The Pearson correlation coefficient (r) values for the LASSO models were 0.83 and 0.64 from the cross-validations for cellular uptake and zeta potential, respectively, and 0.81 and 0.55 from the testing for cellular uptake and zeta potential, respectively. The random forest models performed better with r values 0.88 from the cross-validations for both cellular uptake and zeta potential, and 0.87 and 0.89 from the testing for cellular uptake and zeta potential, respectively. The results showed that QNAR models trained with MD simulation-derived descriptors can accurately predict the properties of gold nanoparticles.

Yan et al. [94] developed universal nanodescriptors by using the Pauling electronegativity to define descriptor characters and the Delaunay tessellation method to simulate the surface chemistry of nanoparticles. Based on the universal nanodescriptors, random forest, and *k* nearest neighbor were employed to develop QNAR models for predicting biological activities and physicochemical properties of gold nanoparticles. Seven gold nanoparticle datasets on enzyme binding affinities, cellular uptake potentials, generation levels of reactive oxygen species, log P, and zeta potential were employed to validate the developed models. The models had good performance with *R*^2^ values 0.59–0.91 in the 5-fold cross-validation and 0.70–0.95 in the external validations, indicating that QNAR models have satisfactory predictability in the prediction of biological activities of nanoparticles.

Deep learning technology such as convolutional neural networks was employed to directly extract nanostructure features from nanoparticle images [95]. Based on features directly learned from nanoparticle images, QNAR models were developed for predicting physicochemical properties (zeta potential and logP) and biological activities (protein adsorption and cellular uptake) of 147 unique nanoparticles, including 123 gold nanoparticles, 12 palladium nanoparticles, and 12 platinum nanoparticles. The convolutional neural network models achieved accurate predictions with all *R*^2^ values larger than 0.68 in the cross-validations and external predictions.

Ensemble learning methods can improve the prediction accuracy of weak models and be employed for constructing QNAR models. Singh et al. [96] reported the first ensemble learning method-based QNAR models for predicting the biological effects of diverse nanoparticles using simple molecular descriptors. Regression and classification models were developed based on five different datasets of nanoparticles including nanoparticles with various metal cores, nanoparticles with similar cores but different surface modifiers, nanoparticles of metal oxide, multi-walled carbon nanotubes with modified surface, and fullerene derivatives. Stochastic gradient boosting and bagging algorithms were incorporated into the models to improve the prediction accuracy of the developed models. 

## 6. Meta-Analysis

Although QNAR can associate the physicochemical properties of ENMs with their toxicity, most of the published QNAR models were developed with small datasets from a single study or a small number of studies, limiting their extrapolations to new ENMs. Meta-analysis, also known as literature data mining, is a method that quantitatively integrates available data and findings from research works on the same topic to generate a comprehensive understanding of the topic [97]. Meta-analysis on toxicity data available for ENMs in the whole body of the literature could generate large high-quality datasets that cover wide nano-structural spaces for developing reliable QNAR models with excellent extrapolation capabilities. A meta-analysis was performed on 17 rodent (rat and mouse) toxicity studies on carbon nanotubes. The meta-analysis resulted in datasets of four pulmonary toxicity endpoints (polymorphonuclear neutrophils, macrophages, lactate dehydrogenase, and total protein) for carbon nanotubes. For each of the carbon nanotubes, toxicity data points were described by 20 experimental conditions and 17 nanoparticle properties (e.g., impurities, physical dimensions, and exposure characteristics) [98]. Regression tree and random forest models were built using 3 to 13 variables selected from the 37 input variables. The goodness-of-fit performance was good with *R*^2^ values 0.62–0.92 for the regression tree models and 0.83–0.95 for the random forest models for the four pulmonary toxicity endpoints. Furthermore, metallic impurities, aggregate size, and carbon nanotube length were found relevant to the rodent pulmonary toxicity endpoints.

In another study, cellular toxicity of cadmium-containing quantum dots was investigated with meta-analysis [99]. In this study, 1741 cell viability-related data points and 24 qualitative and quantitative attributes on material properties and experimental conditions were collected from 307 published papers. Based on these data, random forest regression models were developed and the attributes of quantum dots relevant to cellular toxicity were identified. The models for predicting IC_50_ (exposure concentration corresponding to 50% inhibition of cell growth) reached a coefficient of determination (*R*^2^) up to 0.92. The results demonstrated that cellular toxicity was correlated with quantum dot surface properties, assay type, diameter, and exposure time.

Bial et al. [100] employed a machine learning method, i.e., Bayesian networks to investigate cellular toxicity of cadmium-containing quantum dots based on meta-analysis. They expanded cell viability and IC_50_ datasets from 1741 to 3028 and 514 to 837, respectively. The quantum dots were described using 15 categorical and 3 quantitative attributes. The developed models with 8 most relevant attributes achieved *R*^2^ of 0.81 and 0.85 for predicting cell viability and IC_50_, respectively. The most relevant attributes for predicting cell viability and IC_50_ were diameter, surface ligand, exposure time, shell, surface charge, assay type, surface modification, and concentration. The Bayesian network models were cast as web applications (BN-QDTox) for predicting the cellular toxicity of cadmium-containing quantum dots.

Labouta et al. [101] collected 2896 individual cell viability data points of ENMs along with 17 features including ENM-related parameters (e.g., size, surface charge, concentration, and surface coating material), cell-related attributes (e.g., cell type, age, and morphology), and methodological parameters (e.g., exposure time and cell viability test indicator) from 93 research articles. These data were divided into 3 datasets as data on surface coating material and zeta potential were only available for a part of the nanoparticles. The 3 datasets contained 1052 nanoparticles with known coating material, 1261 nanoparticles with known zeta potential features, and 540 nanoparticles with known coat and zeta potential features. The decision tree algorithm was employed to develop binary classification models based on these 3 datasets and selected features for predicting the cytotoxicity of nanoparticles. In total, four models were developed and their classification accuracies were up to 87.9%, 90%, 88.2%, and 91.8%.

The missing value issue widely exists in meta-analysis datasets. Most of the machine learning algorithms are not able to handle missing values. Thus, methods such as dividing original datasets into subsets and imputation strategies were used to deal with the missing value issue. However, the data size decreases and the reliability of imputed data increases the uncertainty in training models. Association rule mining (ARM) is a rule-based machine learning algorithm, which can deal with incomplete datasets. ARM models were trained on a large cytotoxicity dataset of nanoparticles which contained 4111 samples and 25 qualitative and quantitative attributes from 152 articles, in order to reveal hidden relationships between attributes of nanoparticles and their cytotoxicity [102]. The results revealed that the cytotoxicity of nanoparticles is primarily associated with the core and coating material, synthesis methods, and cell types. Table 3 summarized the recent QNAR and meta-analysis models for predicting toxicological effects of ENMs.

## 7. Concluding Remarks, Challenges, and Perspectives

Computational nanotoxicology models such as MFA, MEM, PBTK, QNAR, and meta-analysis are critical for the environmental risk assessment of ENMs. In this review, the recent progress of computational nanotoxicology models was summarized. A major challenge of MFA and MEM models is the limited parameters to cover realistic environmental fate processes. The current MFA model only estimates the total mass of a specific ENM during the life circle, but cannot provide detailed information on the size, shape, and form of ENMs that are released into the environment. The MFA model can be combined with the MEM model to achieve “source-to-concentration” predictions [103]. However, without detailed input information such as size distribution, the MFA coupled with the MEM model may not enable an accurate prediction. For the MEM model, the lack of mechanistic processes (e.g., agglomeration, transformation) within the model can induce uncertainty in the prediction of the environmental concentration of ENMs. For example, Ag nanoparticles may transform into diverse forms such as AgCl and Ag_2_S and the dissolved Ag can also transform into Ag nanoparticles [104]. The processes can be affected by many environmental factors such as pH, illumination, and dissolved organ matter, increasing the complexity of modeling ENMs’ environmental fate [105]. Thus, a detailed description with proper parameterization on major mechanistic processes can improve the prediction accuracy of ENMs’ environmental concentration. 

With the accurate estimation of ENM concentrations in the environment, the internal exposure concentration of ENMs can be derived by PBTK models. However, most current PBTK models are used for nanomedicine research with intravenous injection as the main exposure route, which is impossible to exist in the environmental media. Ingestion from the mouth and passive uptake via body surface/opening (e.g., anus, skin) may be the main exposure routes to ENMs for organisms in the environment [106]. As protein-corona formation in ENM surface may vary in different exposure routes [107], the toxicokinetics of ENMs in the body can be changed. Thus, new PBTK models of ENMs considering the different exposure routes need to be developed. Parameter acquisition is another challenge for PBTK modeling. With the development of artificial intelligence, some key parameters can be predicted with QNAR models based on machine learning algorithms [108]. However, the models seldom consider the effects of the physiological environment on the physico-chemical properties of ENMs entering into the body. 

The PBTK model can provide a mapping of the dose–response relationship between in vitro and in vivo. With the mapping relation, in vivo toxicity (e.g., hepatotoxicity) can be estimated with in vitro toxicity (e.g., cytotoxicity on hepatocytes), which can be predicted with QNAR models [109,110]. Substantial high-quality data and nanodescriptors are two major challenges for QNAR modeling. Although some databases and datasets were developed for ENMs, the data volume is not enough to meet the standard that makes full use of the potential of machine learning algorithms. For example, a web-based ENMs database was constructed, which contains information on 705 unique ENMs, 11 material types, 6 physicochemical properties, 10 endpoints, and 2142 nanodescriptors [111]. Compared with some public databases for chemicals such as PubChem [112], the ENM database is too small. Unlike small molecules, descriptors for ENMs (i.e., nanodescriptors) are very limited as most nanodescriptors cannot be produced based on the component core. Characteristics such as size, shape, and surface area may be the main differences between two ENMs with the same component core [113]. However, these characteristics cannot be derived quickly, increasing the difficulty of building QNAR models. Thus, methods to easily calculate nanodescriptors still need to be developed.

## Figures and Tables

**Table 1:** Summary of material flow analysis and multimedia environmental models for ENMs.

Model Categories	Methods or Name	ENMs	Environmental Compartments	Technological Compartments	Ref.
Material flow analysis	Deterministic	Ag, TiO_2_, CNT	Air, water, and soil	Waste incineration plants, landfills, and sewage treatment plants	[47]
	Probabilistic	Ag, TiO_2_, ZnO	Air, natural and urban soil, sludge-treated soil, surface water	Production and manufacturing, consumption, recycling, landfill, waste incineration plant	[50]
	Probabilistic	Ag, TiO_2_	Surface water, soil, air	Production, manufacturing, use, wastewater system, solid waste management, export	[51]
	Probabilistic, dynamic	TiO_2_	Air, natural and urban soil, subsurface, sludge-treated soil, surface soil	Production, manufacturing, consumption, in-use stock, release, wastewater management, solid waste management	[52]
	Probabilistic, dynamic	TiO_2_, ZnO, Ag, CNT	Atmosphere, natural and urban soil, sewage sludge treated soil, surface waters, and sediment	Landfills, sewage treatment plants, waste incineration plants, recycling and export	[55]
	Probabilistic, dynamic	TiO_2_, ZnO, Ag, CNT	Air, soil, surface water, and sediment	Production/manufacturing, wastewater treatment, waste incineration, landfill, and recycling	[56]
	Probabilistic, dynamic	CNT, Ag, TiO_2_, ZnO	Air, soil, surface water, and sediment	Production/manufacturing, wastewater treatment, waste incineration, landfill, and recycling	[57]
	Probabilistic, dynamic	SiO_2_, iron oxides, CeO_2_, Al_2_O_3_, quantum dots	Air, soil, water, sediment	Production, manufacturing, sewage treatment plants, waste incineration plants, landfills, export and recycling	[58]
Multimedia environmental models	MendNano	Al_2_O_3_, CeO_2_, CuO, Fe_3_O_4_, TiO_2_, ZnO, Ag, nanoclays, SiO_2_, CNT	Air, water, soil, sediment, biota	-	[60]
	SimpleBox4Nano	TiO_2_	Air, rain, surface waters, soil, and sediment	-	[61]
	nanoFate	CeO_2_, CuO, TiO_2_, and ZnO	Atmosphere, soil, water, sediment	-	[59]
	nanoFate	TiO_2_	Air, freshwater, marine, natural soil, urban soil, agricultural soil, biosolids soil		[62]
	SimpleBox4Nano	CeO_2_, TiO_2_, ZnO	Atmosphere, water, sediment, soil		[63]

**Table 2:** Summary of PBTK models for ENMs.

ENMs	Species	Exposure Routes	Model Structures	Ref.
Gold	Rats	Intraperitoneal injection	Heart, kidneys, muscle, skin, brain, adipose, gonads, liver, stomach, spleen, pancreas, small intestine, large intestine, bone, and lungs (each organ includes 4 sub-compartments: plasma, vascular endothelium, macrophages, and interstitial space)	[68]
Cerium dioxide	Rats	Intravenous administration	Blood, liver, spleen, lung, kidney, heart, brain, bone marrow, and other tissues	[70]
Gold	Rats	Inhalation	Blood, liver, kidney, heart, brain, lymph nodes, spleen, gastrointestinal tract, olfactory, and tracheobronchial	[74]
Gold	Human	Intravenous injection	Liver, venous plasma, kidney, and skin	[76]
Quantum dot	Mice	Intravenous injection	Lung, liver, kidney, spleen, and the rest of the body, blood (each organ includes 3 subcompartments: vascular space, phagocytic cells, and tissue)	[77]
Glycol-coated gold	Mice	Intravenous injection	Blood, liver, spleen, kidneys, lungs, brain, and rest of the body tissues	[78]
Gold	Rats	Intravenous, oral gavage, intratracheal instillation, and endotracheal inhalation	Blood, lungs, liver, spleen, gastrointestinal tract, kidneys, and remaining tissues	[79]
Silver	Mice	Intratracheal instilled	Lung, spleen, kidney, liver, brain, heart, and blood	[80]

**Table 3:** QNAR and meta-analysis models for predicting toxicological effects of ENMs.

Model Categories	ENMs	Endpoints	Algorithms	Data Size	Descriptors	Performance	Ref.
	TiO_2_	Cytotoxicity	Linear regression	34	First generation of simple periodic table-based descriptors	*R*^2^ = 0.922–0.926	[90]
	Metal oxide	Cytotoxicity	Multiple linear regression	12	Second generation of simple periodic table-based descriptors	*R*^2^ = 0.88	[91]
	Gold	Cell uptake	*k* nearest neighbors	34	Theoretical descriptors based on virtual structures	*R*^2^ > 0.918	[92]
	Gold	Cell uptake	LASSO and random forest regression	154	Simulation-derived descriptors	*r* = 0.9	[93]
	Gold	(1) Enzyme binding affinities; (2) cellular uptake; (3) cellular uptake.	Random forest and *k* nearest neighbor	(1) 47; (2) 41; (3) 71	Universal nanodescriptors	(1) *R*^2^cv = 0.9; (2) *R*^2^cv = 0.92; (3) *R*^2^cv = 0.84.	[94]
	Gold; platinum; palladium.	Cellular uptake and protein adsorption	Convolutional neural network	147	Nanoparticle images	*R*^2^ > 0.68	[95]
	(1) Metal cores; (2) metal; (3) metal oxide; (4) carbon nanotubes.	(1) ATP content, apoptosis, mitochondrial membrane potential; (2) cell uptake; (3) bacteria cytotoxicity; (4) cell cytotoxicity.	Ensemble learning	(1) 51; (2) 109; (3) 17; (4) 80	Chemistry Development Kit, ChemSpider	(1) CCC = 0.961; (2) CCC = 0.932; (3) CCC = 0.974; (4) CCC = 0.932.	[96]
Meta-analysis	Carbon nanotube	Pulmonary toxicity	Random forest	136	20 experimental conditions; 17 nanoparticle properties; 4 experimental endpoints.	No data	[98]
	Cadmium quantum dots	Cellular toxicity	Random forest	1741	24 qualitative/quantitative attributes on material properties and experimental conditions	*R*^2^ = 0.92	[99]
	Cadmium quantum dots	Cellular toxicity	Bayesian network	3028	15 categorical and 3 quantitative attributes, including quantum dot properties, surface properties, experimental conditions, and biological conditions	*R*^2^ > 0.81	[100]
	Organic and inorganic nanoparticles	Cytotoxicity	Decision trees	2896	Nanoparticle-related features, cell-related features, methodological parameters	ACC > 87.9%	[101]
	Inorganic, organic, and carbon based	Cytotoxicity	Association rule mining	4111	15 qualitative and 10 quantitative attributes	No data	[102]
